# Associations between cognitive performance and Mediterranean dietary pattern in patients with type 1 or type 2 diabetes mellitus

**DOI:** 10.1038/s41387-020-0111-z

**Published:** 2020-04-01

**Authors:** Theresa Kössler, Katharina S. Weber, Wolfgang Wölwer, Annika Hoyer, Klaus Strassburger, Volker Burkart, Julia Szendroedi, Michael Roden, Karsten Müssig, for the GDS Group, M. Roden, H. Al-Hasani, A. E. Buyken, B. Belgardt, G. Geerling, C. Herder, A. Icks, J. Kotzka, O. Kuß, E. Lammert, J-H. Hwang, K. Müssig, D. Markgraf, W. Rathmann, J. Szendroedi, D. Ziegler

**Affiliations:** 1 Institute for Clinical Diabetology, German Diabetes Center, Leibniz Center for Diabetes Research at Heinrich Heine University Düsseldorf, Düsseldorf, Germany; 2 Division of Endocrinology and Diabetology, Medical Faculty, Heinrich Heine University, Düsseldorf, Germany; 3 German Center for Diabetes Research (DZD), München-Neuherberg, Germany; 4 Institute of Epidemiology, University of Kiel, University Hospital Schleswig-Holstein, Kiel, Germany; 5 Department of Psychiatry and Psychotherapy, Medical Faculty, Heinrich Heine University Düsseldorf, Düsseldorf, Germany; 6 Institute for Biometrics and Epidemiology, German Diabetes Center, Leibniz Center for Diabetes Research at Heinrich Heine University Düsseldorf, Düsseldorf, Germany

**Keywords:** Lifestyle modification, Risk factors, Nutrition

## Abstract

Diabetes mellitus has been associated with impaired cognitive performance, particularly in verbal memory. Mediterranean diets (MedD) may lead to improvements in overall and single cognitive functions. We hypothesised that adherence to MedD associates with better performance in verbal memory in patients with type 1 or type 2 diabetes. Thus, we performed a cross-sectional analysis including patients with recently diagnosed type 1 (*n* = 75) or type 2 diabetes (*n* = 118), metabolically healthy individuals (*n* = 41) and individuals with type 1 (*n* = 44) or type 2 diabetes (*n* = 62) of at least five years after diagnosis. Participants underwent comprehensive metabolic phenotyping and cognitive testing. Adherence to the Modified Mediterranean diet scale (MMDS) was computed from a food frequency questionnaire. Among patients with type 2 diabetes with a known diabetes duration ≥5 years, closer adherence to the MMDS was associated with higher score in verbal memory after adjustment for potential confounders (*P* = 0.043). Adherence to the MMDS did not relate to verbal memory in recently diagnosed type 2 diabetes (*P* = 0.275), recently diagnosed or longer-standing type 1 diabetes (*P* = 0.215 and *P* = 0.626, respectively) or metabolically healthy individuals (*P* = 0.666). In conclusion, closer adherence to MedD may exert beneficial effects on cognitive performance in the course of type 2 diabetes.

## Introduction

Diabetes mellitus has been associated with an increased risk of cognitive impairment^1^. A systematic review on dietary behaviour and cognitive function found that the Mediterranean diet (MedD), which is characterized by high intake of fruits, vegetables, cereals, nuts and olive oil, moderate intake of fish, wine and dairy products and low intake of saturated fat and meat, may have beneficial effects on cognitive performance^2^. Furthermore, individuals following a MedD particularly feature improvements in single cognitive domains, i.e., memory and fluency^3^. However, the majority of these studies focused on MedD and cognitive performance in healthy elderly individuals or individuals with increased cardiovascular risk, including diabetes as one potential risk factor. The limited number of studies focusing solely on patients with type 2 diabetes show inconsistent findings. While one study shows that higher adherence to MedD was associated with lower risk of cognitive impairment^4^, other studies only observe this association in individuals without diabetes^2^. Therefore, the effect of MedD on cognitive function in individuals with diabetes needs further investigation. Moreover, it is still unclear whether the association of MedD with cognitive performance differs between diabetes types and if so whether such differences occur already early during the course of diabetes. In a previous study, we showed an association between verbal memory and metabolic parameters in patients with type 2 diabetes^1^. The aim of the present study was (i) to identify associations between MedD and cognitive performance, particularly in verbal memory, in patients with recently diagnosed type 1 or type 2 diabetes or a known diabetes duration of at least five years as well as in metabolically healthy individuals and (ii) to investigate possible differences between these diabetes types.

## Materials and methods

The present cross-sectional analysis comprises participants of the German Diabetes Study (GDS)^5^, namely 41 metabolically healthy individuals, 119 individuals with type 1 diabetes and 180 individuals with type 2 diabetes with a known disease duration of <1 year or ≥5 years. Patients with type 1 diabetes were treated with insulin. In contrast patients with type 2 diabetes were treated with oral glucose-lowering drugs in 19% of cases (14% metformin, 3% dipeptidyl dipeptidase-4 inhibitors, 1% sulfonylureas, 1% unknown), with insulin in 6% of cases and only lifestyle modification in 75% of cases. By definition, none of the metabolically healthy humans received any glucose-lowering medication. Participants were consecutively included if they underwent cognition tests and entirely completed the Multiple Choice Word Test (MWT)-B and the food frequency questionnaire (FFQ). All participants gave written informed consent to the study, which was approved by the ethics board of Heinrich Heine University Düsseldorf, Germany.

Participants underwent comprehensive metabolic phenotyping and cognition testing using the standardized test battery “Brief Assessment of Cognition in Schizophrenia” (BACS) and additional cognitive tests assessing different cognitive domains as described^1^.

Habitual dietary intake during the last 12 months was assessed using the self-administered 148 food and beverage items semi-quantitative FFQ, designed and validated within the European Prospective Investigation into Cancer and Nutrition (EPIC) Potsdam study^5,6^. Mean total energy intake (TEI) (MJ/d) and food intake (g/d) were derived for each participant and the Modified Mediterranean diet scale (MMDS) was applied^7^. The MMDS indicates the degree of adherence to the traditional MedD by assigning a value of 0 or 1 to nine components, with the use of the sex- and cohort-specific median as cut-off. For persons whose consumption was at or above the median, a value of 1 was assigned for presumed beneficial components (vegetables, legumes, fruits, cereals, fish) and a value of 0 for presumed detrimental components (meat, dairy products). For ethanol, a value of 1 was assigned for a moderate alcohol consumption. Individuals with a lipid ratio (unsaturated to saturated fatty acids) at or above the median were assigned a value of 1. The MMDS ranges from 0 (minimal adherence) to 9 (maximal adherence)^7^.

SAS (version 9.4; SAS Institute, Cary, NC) procedures were used for data analyses. *P* < 0.05 was considered statistically significant. Results are presented as mean ± standard deviation (SD) for normally and median (25th; 75th percentiles) for non-normally distributed data. Scores of MMDS were used as independent predictors in multiple linear regression models with cognition test results as dependent variables. Model 1 presents unadjusted data. Model 2 considered age, sex and TEI as covariates. Model 3 was additionally adjusted for crystallized intelligence (MWT-B). In addition, fasting C-peptide was added to model 3 in order to adjust for insulin secretion. For better illustration of effect sizes, adjusted means of the dependent variables were calculated by tertiles of the MMDS. Interaction of the MMDS and diabetes type on verbal memory was investigated using multiple linear regression analysis, adjusted for the respective confounders of model 3.

## Results

Metabolically healthy individuals and patients with type 2 diabetes were nominally older and more overweight/obese than patients with type 1 diabetes. Metabolic control of all patients with diabetes based on HbA1c was within the range recommended by current guidelines (Table 1)^8^.

**Table 1:** Characteristics of the study participants.

	Metabolically healthy individuals (*n* = 41)	Individuals with recently diagnosed diabetes (*n* = 193)		Individuals with a known diabetes duration of ≥5 years (*n* = 106)	
Variables		Type 1 diabetes	Type 2 diabetes	Type 1 diabetes	Type 2 diabetes
*N* (% male)	41 (85)	75 (59)	118 (64)	44 (61)	62 (58)
Age [years]	49.5 ± 12.1	35.9 ± 10.5	52.2 ± 8.8	43.0 ± 13.5	59.7 ± 9.7
BMI [kg/m^2^]	29.1 ± 5.9	25.2 ± 4.0	32.2 ± 5.7	25.2 ± 2.9	31.5 ± 5.7
Duration since diagnosis of diabetes [months]	–	6.1 ± 3.0	5.6 ± 3.3	72.0 ± 15.0	74.1 ± 18.3
HbA1c [%]	5.3 ± 0.3	6.3 ± 1.0	6.5 ± 0.9	6.9 ± 1.0	6.9 ± 1.1
HbA1c [mmol/mol]	34 ± 3	45 ± 11	48 ± 10	52 ± 11	52 ± 12
Fasting C-peptide [ng/ml]	2.0 [1.4; 2.5]	0.9 [0.5; 1.3]	3.0 [2.4; 3.9]	0.3 [0.1; 0.8]	2.8 [2.2; 3.9]
Triglycerides [mg/dl]	93 [76; 148]	76 [55; 109]	129 [97; 199]	76 [55; 109]	177 [108; 240]
Total cholesterol [mg/dl]	203 [181; 225]	178 [159; 195]	195 [178; 226]	178 [159; 195]	202 [177; 235]
HDL-cholesterol [mg/dl]	60 [47; 70]	59 [49; 69]	45 [37; 52]	59 [49; 69]	49 [41; 59]
LDL-cholesterol [mg/dl]	133 [108; 150]	105 [87; 124]	132 [107; 156]	105 [87; 124]	128 [106; 158]

Data are *n* (%), mean ± SD or median (25th; 75th percentile).

*HDL* high density lipoprotein, *LDL* low-density lipoprotein.

Mean values of cognition tests in healthy individuals and diabetes patients were within the normal range (Supplementary Table 1). At times of diagnosis and about 5 years after diagnosis, patients with type 1 diabetes and healthy individuals had an estimated TEI of about 10 MJ/d, whereas patients with type 2 diabetes indicated to consume about 8.6 MJ/d. Overall, participants reached a mean MMDS of about 4.5 (Supplementary Table 2).

Among individuals with type 2 diabetes at ≥5 years after diagnosis, closer adherence to MMDS was associated with higher score in verbal memory, while neither for recently diagnosed type 2 diabetes, type 1 diabetes nor metabolically healthy individuals, adherence to MMDS was related to verbal memory (Table 2). Results did not change upon additional adjusting for fasting C-peptide (individuals with type 2 diabetes at ≥5 years after diagnosis (mean 95% confidence interval for tertile (T) 1, T2 and T3 of MMDS adherence): T1: −0.66 (−1.22; −0.11), T2: −0.87 (−1.27; −0.46), T3: −0.07 (−0.56; 0.41), *P* = 0.042). Interaction analyses revealed that the association between MMDS and verbal memory only holds true for patients with type 2 diabetes with a known diabetes duration of at least 5 years (*P* = 0.024).

**Table 2:** Associations between the Modified Mediterranean diet scale and verbal memory.

	T1	T2	T3	
	Mean (95% CI)	Mean (95% CI)	Mean (95% CI)	*P*^*a*^
Metabolically healthy individuals (*n* = 41)				
Model 1^b^	0.19 (−0.57; 0.95)	0.59 (0.06; 1.11)	−0.20 (−0.96; 0.56)	0.396
Model 2^c^	0.53 (−0.21; 1.27)	0.99 (0.36; 1.61)	0.26 (−0.49; 1.01)	0.861
Model 3^d^	0.56 (−0.11; 1.23)	0.84 (0.27; 1.42)	0.27 (−0.40; 0.95)	0.666
Individuals with recently diagnosed diabetes				
Type 1 diabetes (*n* = 75)				
Model 1^b^	0.23 (−0.42; 0.88)	0.23 (−0.25; 0.71)	0.46 (−0.16; 1.08)	0.355
Model 2^c^	0.01 (−0.65; 0.67)	0.23 (−0.24; 0.71)	0.42 (−0.18; 1.03)	0.272
Model 3^d^	0.00 (−0.63; 0.64)	0.23 (−0.22; 0.69)	0.41 (−0.17; 0.99)	0.215
Type 2 diabetes (*n* = 118)				
Model 1^b^	−0.40 (−0.78; −0.01)	−0.28 (−0.55; 0.00)	−0.11 (−0.50; 0.27)	0.207
Model 2^c^	−0.31 (−0.70; 0.07)	−0.23 (−0.51; 0.05)	−0.17 (−0.57; 0.23)	0.385
Model 3^d^	−0.35 (−0.71; 0.01)	−0.22 (−0.48; 0.03)	−0.18 (−0.55; 0.19)	0.275
Individuals with a known diabetes duration of ≥5 years				
Type 1 diabetes (*n* = 44)				
Model 1^b^	0.33 (−0.20; 0.86)	0.15 (−0.30; 0.61)	0.72 (0.12; 1.32)	0.395
Model 2^c^	0.33 (−0.16; 0.82)	0.32 (−0.08; 0.72)	0.52 (0.00; 1.04)	0.731
Model 3^d^	0.32 (−0.16; 0.80)	0.33 (−0.06; 0.73)	0.52 (0.00; 1.03)	0.626
Type 2 diabetes (*n* = 61)				
Model 1^b^	−0.84 (−1.44; −0.24)	−0.74 (−1.17; −0.32)	0.01 (−0.50; 0.53)	**0.010**
Model 2^c^	−0.78 (−1.38; −0.17)	−0.82 (−1.25; −0.38)	−0.03 (−0.54; 0.49)	**0.026**
Model 3^d^	−0.76 (−1.35; −0.17)	−0.78 (−1.21; −0.35)	−0.10 (−0.61; 0.41)	**0.043**

Values are least-square means with 95% CI.

Bold indicates *P* < 0.05.

*MWT-B* multiple choice word test B, *T* tertile, *TEI* total energy intake.

^a^Based on multiple linear regression models with the Modified Mediterranean diet scale as continuous variable.

^b^Model 1, unadjusted.

^c^Model 2 adjusted for age, sex and TEI.

^d^Model 3 adjusted for model 2 plus MWT-B.

There was no association between adherence to MMDS and further parameters of cognitive performance in this study population (Supplementary Table 3).

## Discussion

These data suggest that closer adherence to MedD was associated with better performance in verbal memory in patients with type 2 diabetes with known diabetes duration ≥5 years, but not in patients with recently diagnosed type 2 diabetes or in patients with type 1 diabetes or metabolically healthy individuals.

The MedD has been already reported to exert beneficial effects on cardiovascular disease and additionally on cognitive performance mainly in healthy elderly individuals or individuals with increased cardiovascular risk^2,9,10^. The present results show an association between MedD and verbal memory in individuals with diabetes. Although the underlying mechanisms are currently unknown, one may speculate that the high content of antioxidants in MedD may contribute to better cognitive performance by reducing the production of reactive oxygen species and attenuating inflammatory processes, both of which have been linked to cognitive decline^10^. Furthermore, positive effects might be mediated by n-3 fatty acids (FA). Higher dietary n-3 FA intake or circulating blood n-3 FA levels have been associated with better global or single cognitive function^11,12^, which was mainly explained by their anti-inflammatory, antioxidative and antithrombotic properties^13^. However, the influence of n-3 FA is controversial according to the literature, as not all studies observed beneficial effects, possibly due to different study designs, methods and varying quality of studies^11,14^.

Interestingly, our analysis did not find associations between MedD and cognitive performance in patients with type 1 diabetes. In our previous study, impaired verbal memory was observed only in patients with type 2 diabetes ≥5 years after diagnosis but not in type 1 diabetes^1^. We also observed beneficial effects on memory function only among patients with diabetes ≥5 years after diagnosis, which is consistent with other prospective studies showing positive associations of MedD with cognitive function after 3–7.6 years of follow-up^2^. Our cohort primarily include metabolically well-controlled patients with diabetes, which is in line with a study showing improved overall cognitive function after following MedD only in patients with type 2 diabetes with HbA1c below 7.0 %^4^.

While the majority of studies showed beneficial effects of MedD on global cognition^2,15^, we only observed associations with improved verbal memory. This corresponds to studies showing better memory function after following MedD supplemented with olive oil or nuts compared to a low-fat control diet in patients at high cardiovascular risk^3,16^. The hippocampus mainly controls memory function and appears to be very sensitive to external factors, to which MedD could have contributed^10^.

However, not all studies observed associations between MedD and cognitive performance or single cognitive domains^9^, which may be due to the heterogeneity of methods used to assess cognitive function and dietary intake. In order to increase comparability with previous studies, we used a generally accepted score to define adherence to MedD^7,15^.

Strengths of our study are the comprehensive metabolic phenotyping and neurocognitive testing, comprising multiple cognitive domains. Besides metabolically healthy individuals, we included patients with type 1 and type 2 diabetes, in contrast to previous studies which did not distinguish between diabetes types^2,10,16^. However, given that our data do not include individual follow-up data, but one group with recent onset diabetes and another group with diabetes duration of ≥5 years, we cannot validate associations of MedD with cognitive performance during the course of diabetes. Another limitation is the use of an FFQ to assess food intake, which has been validated and used in larger cohorts. However, in order to address this issue, we studied a predefined dietary pattern, thus limiting the analyses to the food level and only classifying participants into low and high consumers. Furthermore, we did not consider the glycemic load of various food groups like fruits and vegetables as potential confounder in our analysis as the EPIC-FFQ does not allow the assessment of this variable. Further limitations are the relatively small sample size of the different cohorts, which did not allow matching the groups for factors known to influence cognitive function, such as age, sex or socio-economic factors. However, all participants showed a comparable good metabolic control and results were adjusted for risk factors potentially confounding associations between MedD and cognitive performance.

In conclusion, higher adherence to MedD is associated with better verbal memory in patients with a known duration of type 2 diabetes of at least five years, but not in patients with type 1 diabetes.

## Supplementary information

Unadjusted variables of cognition test results of metabolically healthy individuals and individuals with type 1 and type 2 diabetes recently diagnosed as well as ≥5 years after diagnosis.

Dietary intake of individuals with type 1 and type 2 diabetes recently diagnosed as well as ≥5 years after diagnosis and metabolically healthy individuals.

Associations between the Modified Mediterranean diet scale and cognition test results.

## Data Availability

A request and transfer process has been established so that researches may apply for data by contacting the study coordinators via email (GDS@ddz.de). Once approved by the steering committee, the requesting researcher and the principal investigator of GDS sign a contract on the terms and conditions of data transfer and transmission of results back to the German Diabetes Center.
