# Supplementary material for: Associations between cognitive performance and Mediterranean dietary pattern in patients with type 1 or type 2 diabetes mellitus
Source: Nutr Diabetes. 2020 Apr 1;10:10. doi: 10.1038/s41387-020-0111-z (PMC7113267; doi:10.1038/s41387-020-0111-z)
Supplement: Supplementary file 1 — Unadjusted variables of cognition test results of metabolically healthy individuals and individuals with type 1 and type 2 diabetes recently diagnosed as well as ≥5 years after diagnosis. [file 41387_2020_111_MOESM1_ESM.docx]

**Supplementary Table 1:** Unadjusted variables of cognition test results of metabolically healthy individuals and individuals with type 1 and type 2 diabetes recently diagnosed as well as ≥5 years after diagnosis.

|  | Metabolically healthy individuals (n=41) | Individuals with recently diagnosed diabetes (n=193) | | Individuals with a known diabetes duration of ≥5 years (n=106) | |
| --- | --- | --- | --- | --- | --- |
| Variables |  | Type 1 diabetes | Type 2 diabetes | Type 1 diabetes | Type 2 diabetes |
| Verbal memory^†,*^ | 0.3±1.2 | 0.3±1.4 | -0.3±1.1 | 0.4±1.0 | -0.5±1.2 |
| Digit sequencing^†,$^ | 0.2±0.9 | -0.1±1.0 | -0.1±1.1 | -0.1±1.0 | 0.0±1.1 |
| Token motor task^†,*^ | 0.4±1.0 | 0.4±0.9 | 0.3±1.0 | 0.5±0.9 | 0.0±1.2 |
| Verbal fluency^†^ | 0.0±1.1 | 0.0±1.2 | 0.0±1.1 | 0.0±1.0 | -0.3±1.0 |
| Symbol coding score^†^ | -0.2±0.8 | 0.0±0.9 | -0.3±1.1 | 0.0±0.9 | -0.5±1.1 |
| Tower of London^†^ | 0.1±0.8 | 0.4±0.8 | 0.1±0.8 | 0.2±0.7 | -0.1±1.0 |
| BACS composite score^†,§^ | 0.2±0.9 | 0.3±1.0 | -0.1±1.0 | 0.2±0.9 | -0.4±1.0 |
| TMT_A^‡^ | 49.7±8.9 | 51.8±10.3 | 49±11.2 | 50.1±9.9 | 49.0±8.8 |
| TMT_B^‡,¥^ | 52.0±9.2 | 52.7±9.6 | 50.9±9.9 | 49.5±8.8 | 49.0±8.7 |
| Pictures of facial affect^‡,§^ | 41.9±10.4 | 46.9±9.9 | 44.2±11.4 | 45.0±9.8 | 43.1±9.4 |
| MWT-B^#^ | 115.1±12.1 | 115.7±13.8 | 118.7±15.0 | 117±12.5 | 113.4±12.8 |

Data are ^†^mean±SD of z-score values, ^‡^mean±SD of *T*-score values or ^#^mean±SD of IQ values. Given that few participants failed to finish all cognitive tests, data are only available for ^*^n=61 patients with a known type 2 diabetes duration ≥5 years, ^$^n=74 and n=117 patients with recently diagnosed type 1 and type 2 diabetes, respectively, ^§^n=74 and n=117 patients with recently diagnosed type 1 and type 2 diabetes, respectively and n=60 patients with a known type 2 diabetes duration >5 years, ^¥^n=117 patients with recently diagnosed and n=60 patients with a known type 2 diabetes duration ≥5 years and ^§^n=117 patients with recently diagnosed type 2 diabetes.

BACS, Brief Assessment of Cognition in Schizophrenia. MWT-B, multiple choice word test B. TMT_A/B, trail making test A/B.
