# Supplementary material for: Associations between cognitive performance and Mediterranean dietary pattern in patients with type 1 or type 2 diabetes mellitus
Source: Nutr Diabetes. 2020 Apr 1;10:10. doi: 10.1038/s41387-020-0111-z (PMC7113267; doi:10.1038/s41387-020-0111-z)
Supplement: Supplementary file 2 — Dietary intake of individuals with type 1 and type 2 diabetes recently diagnosed as well as ≥5 years after diagnosis and metabolically healthy individuals. [file 41387_2020_111_MOESM2_ESM.docx]

**Supplementary Table 2**: Dietary intake of individuals with type 1 and type 2 diabetes recently diagnosed as well as ≥5 years after diagnosis and metabolically healthy individuals.

|  | Metabolically healthy individuals (n=41) | Individuals with recently diagnosed diabetes (n=193) | | Individuals with a known diabetes duration of ≥5 years (n=106) | |
| --- | --- | --- | --- | --- | --- |
| Variables |  | Type 1 diabetes | Type 2 diabetes | Type 1 diabetes | Type 2 diabetes |
| Total energy intake [MJ/d] | 10.1 (8.1; 12.5) | 10.0 (8.6; 12.2) | 8.7 (7.1; 11.5) | 9.7 (8.5; 13.1) | 8.5 (6.9; 10.6) |
| Modified Mediterranean diet scale | 4.54+1.61 | 4.57+1.60 | 4.59+1.56 | 4.45+1.56 | 4.63+1.52 |
| Vegetables [g/d] | 190.4 (152.1; 229.7) | 219.8 (178.6; 268.7) | 225.2 (151.7; 278.9) | 184.6 (144.3; 257.5) | 201.2 (162.3; 286.9) |
| Legumes [g/d] | 1.82 (1.82; 6.81) | 1.82 (1.20; 6.81) | 1.82 (1.82; 6.81) | 1.82 (1.82; 6.81) | 1.82 (1.82; 6.81) |
| Fruits [g/d] | 184.5 (114; 260.9) | 154.2 (90.4; 211.0) | 164.1 (88.9; 258.9) | 183.5 (114.4; 248) | 187.4 (123.5; 314) |
| Cereals [g/d] | 195.4 (117.9; 283.1) | 178.0 (139.5; 301.0) | 157.7 (112.2; 224.5) | 168.4 (135.5; 226.6) | 138.5 (119.4; 164.2) |
| Fish [g/d] | 19.6 (7.3; 56.9) | 32.2 (7.3; 56.0) | 19.8 (7.3; 44.4) | 19.8 (7.3; 32.2) | 19.6 (7.3; 44.4) |
| Meat [g/d] | 127.3 (85.1; 210.4) | 142.3 (84.8; 195.7) | 126.7 (83.6; 197.9) | 126.5 (86.0; 187.5) | 113.4 (77.3; 177.2) |
| Dairy [g/d] | 221.2 (123.2; 299.6) | 242.4 (156.7; 382.2) | 206.1 (123.9; 313.7) | 265.4 (184.1; 384.2) | 186.6 (116.4; 322) |
| Ethanol [g/d] | 7.09 (2.44; 24.15) | 8.48 (2.51; 19.69) | 4.47 (1.88; 12.31) | 5.17 (2.43; 15.41) | 3.39 (1.13; 11.57) |
| Saturated fatty acids [g/d] | 41.8 (35.2; 53.2) | 43.7 (35.1; 56.0) | 38.2 (29.2; 47.8) | 44.6 (37.4; 54.1) | 36.1 (29.3; 44.6) |
| Monounsaturated fatty acids [g/d] | 39.4 (31.7; 46.3) | 42.6 (36.1; 53.0) | 36.3 (27.4; 49.2) | 42.6 (36.2; 51.5) | 34.7 (27.2; 46.9) |
| Polyunsaturated fatty acids [g/d] | 18.9 (15.2; 23.0) | 20.2 (16.7; 26.4) | 18.5 (13.8; 24.0) | 20.5 (15.3; 27.1) | 19.4 (14.9; 25.8) |

Data are mean±SD or median (P_25_; P_75_).
