# Supplementary material for: Associations between cognitive performance and Mediterranean dietary pattern in patients with type 1 or type 2 diabetes mellitus
Source: Nutr Diabetes. 2020 Apr 1;10:10. doi: 10.1038/s41387-020-0111-z (PMC7113267; doi:10.1038/s41387-020-0111-z)
Supplement: Supplementary file 3 — Associations between the Modified Mediterranean diet scale and cognition test results. [file 41387_2020_111_MOESM3_ESM.docx]

**Supplementary Table 3:** Associations between the Modified Mediterranean diet scale and cognition test results.

|  | Metabolically healthy individuals | | Individuals with recently diagnosed diabetes | | | | Individuals with a known diabetes duration of ≥5 years | | | |
| --- | --- | --- | --- | --- | --- | --- | --- | --- | --- | --- |
|  |  |  | Type 1 diabetes | | Type 2 diabetes | | Type 1 diabetes | | Type 2 diabetes | |
|  | β (95% CI) | *P** | β (95% CI) | *P** | β (95% CI) | *P** | β (95% CI) | *P** | β (95% CI) | *P** |
| Digit sequencing^#^ |  |  |  |  |  |  |  |  |  |  |
| Model 1 | -0.03 (-0.21; 0.16) | 0.786 | -0.03 (-0.18; 0.11) | 0.645 | -0.07 (-0.20; 0.05) | 0.258 | 0.01 (-0.19; 0.22) | 0.887 | -0.02 (-0.22; 0.17) | 0.799 |
| Model 2^†^ | 0.01 (-0.18; 0.20) | 0.917 | -0.03 (-0.18; 0.12) | 0.696 | -0.09 (-0.21; 0.04) | 0.183 | 0.02 (-0.21; 0.25) | 0.848 | -0.04 (-0.24; 0.16) | 0.695 |
| Model 3^‡^ | 0.01 (-0.18; 0.20) | 0.933 | -0.02 (-0.17; 0.12) | 0.738 | -0.08 (-0.21; 0.04) | 0.196 | 0.03 (-0.20; 0.26) | 0.774 | -0.09 (-0.27; 0.09) | 0.327 |
| Token motor task^$^ |  |  |  |  |  |  |  |  |  |  |
| Model 1 | 0.11 (-0.09; 0.30) | 0.279 | 0.00 (-0.13; 0.13) | 0.982 | 0.01 (-0.11; 0.13) | 0.867 | 0.17 (-0.01; 0.34) | 0.057 | 0.03 (-0.17; 0.23) | 0.766 |
| Model 2^†^ | 0.05 (-0.17; 0.27) | 0.629 | -0.01 (-0.13; 0.12) | 0.912 | -0.02 (-0.14; 0.10) | 0.754 | 0.18 (-0.02; 0.37) | 0.080 | 0.00 (-0.2; 0.21) | 0.975 |
| Model 3^‡^ | 0.06 (-0.16; 0.28) | 0.599 | -0.01 (-0.13; 0.12) | 0.934 | -0.02 (-0.13; 0.1) | 0.787 | 0.17 (-0.03; 0.38) | 0.086 | 0.00 (-0.21; 0.21) | 0.992 |
| Verbal fluency |  |  |  |  |  |  |  |  |  |  |
| Model 1 | 0.00 (-0.21; 0.22) | 0.981 | 0.07 (-0.10; 0.24) | 0.398 | -0.04 (-0.17; 0.08) | 0.482 | -0.03 (-0.22; 0.16) | 0.767 | 0.07 (-0.10; 0.24) | 0.400 |
| Model 2^†^ | 0.01 (-0.22; 0.24) | 0.923 | 0.06 (-0.11; 0.23) | 0.477 | -0.02 (-0.14; 0.11) | 0.793 | -0.06 (-0.28; 0.17) | 0.605 | 0.07 (-0.11; 0.24) | 0.455 |
| Model 3^‡^ | 0.00 (-0.23; 0.22) | 0.973 | 0.07 (-0.09; 0.23) | 0.402 | -0.01 (-0.14; 0.11) | 0.863 | -0.03 (-0.24; 0.18) | 0.744 | 0.02 (-0.13; 0.17) | 0.803 |
| Symbol coding score |  |  |  |  |  |  |  |  |  |  |
| Model 1 | -0.14 (-0.29; 0.02) | 0.087 | 0.00 (-0.13; 0.14) | 0.962 | 0.00 (-0.12; 0.13) | 0.949 | -0.07 (-0.25; 0.11) | 0.431 | 0.03 (-0.16; 0.22) | 0.765 |
| Model 2^†^ | -0.12 (-0.29; 0.06) | 0.182 | 0.00 (-0.13; 0.14) | 0.951 | -0.04 (-0.17; 0.09) | 0.511 | -0.04 (-0.23; 0.16) | 0.704 | 0.03 (-0.17; 0.23) | 0.771 |
| Model 3^‡^ | -0.13 (-0.30; 0.05) | 0.144 | 0.01 (-0.13; 0.14) | 0.915 | -0.03 (-0.15; 0.09) | 0.578 | -0.02 (-0.20; 0.17) | 0.852 | 0.01 (-0.19; 0.21) | 0.920 |
| Tower of London |  |  |  |  |  |  |  |  |  |  |
| Model 1 | 0.07 (-0.08; 0.22) | 0.369 | 0.00 (-0.12; 0.13) | 0.949 | -0.08 (-0.18; 0.02) | 0.130 | 0.05 (-0.09; 0.18) | 0.493 | -0.11 (-0.28; 0.05) | 0.170 |
| Model 2^†^ | 0.11 (-0.05; 0.26) | 0.171 | 0.01 (-0.12; 0.13) | 0.905 | -0.10 (-0.20; 0.00) | 0.054 | 0.12 (-0.02; 0.27) | 0.095 | -0.10 (-0.27; 0.07) | 0.235 |
| Model 3^‡^ | 0.10 (-0.05; 0.26) | 0.191 | 0.01 (-0.11; 0.13) | 0.830 | -0.10 (-0.19; 0.00) | 0.060 | 0.13 (-0.02; 0.27) | 0.087 | -0.09 (-0.26; 0.08) | 0.292 |
| BACS composite score^§^ |  |  |  |  |  |  |  |  |  |  |
| Model 1 | -0.02 (-0.21; 0.17) | 0.809 | 0.03 (-0.12; 0.18) | 0.693 | -0.02 (-0.14; 0.09) | 0.678 | 0.07 (-0.10; 0.25) | 0.413 | 0.06 (-0.12; 0.24) | 0.520 |
| Model 2^†^ | 0.02 (-0.17; 0.20) | 0.835 | 0.03 (-0.12; 0.18) | 0.690 | -0.05 (-0.17; 0.07) | 0.419 | 0.08 (-0.11; 0.28) | 0.396 | 0.04 (-0.15; 0.23) | 0.669 |
| Model 3^‡^ | 0.01 (-0.17; 0.19) | 0.939 | 0.04 (-0.10; 0.17) | 0.577 | -0.04 (-0.15; 0.07) | 0.455 | 0.10 (-0.09; 0.29) | 0.282 | 0.00 (-0.18; 0.17) | 0.969 |
| TMT_A |  |  |  |  |  |  |  |  |  |  |
| Model 1 | -0.95 (-2.71; 0.80) | 0.279 | 0.34 (-1.17; 1.84) | 0.658 | 0.48 (-0.84; 1.80) | 0.475 | -0.65 (-2.61; 1.32) | 0.512 | 0.50 (-0.98; 1.98) | 0.503 |
| Model 2^†^ | -0.97 (-2.90; 0.96) | 0.314 | 0.35 (-1.17; 1.88) | 0.645 | 0.10 (-1.24; 1.43) | 0.884 | -0.16 (-2.42; 2.11) | 0.890 | 0.52 (-1.01; 2.06) | 0.497 |
| Model 3^‡^ | -0.98 (-2.95; 0.98) | 0.317 | 0.42 (-1.06; 1.89) | 0.575 | 0.14 (-1.19; 1.46) | 0.837 | 0.05 (-2.13; 2.23) | 0.966 | 0.33 (-1.18; 1.83) | 0.664 |
| TMT_B^¥^ |  |  |  |  |  |  |  |  |  |  |
| Model 1 | -0.06 (-1.90; 1.78) | 0.950 | -0.48 (-1.87; 0.91) | 0.496 | 0.15 (-1.02; 1.32) | 0.796 | 0.99 (-0.73; 2.72) | 0.252 | 0.35 (-1.14; 1.85) | 0.638 |
| Model 2^†^ | 0.40 (-1.63; 2.44) | 0.691 | -0.56 (-1.96; 0.84) | 0.426 | 0.02 (-1.20; 1.23) | 0.974 | 1.34 (-0.68; 3.35) | 0.187 | 0.54 (-1.04; 2.12) | 0.497 |
| Model 3^‡^ | 0.30 (-1.71; 2.31) | 0.767 | -0.51 (-1.88; 0.86) | 0.457 | 0.08 (-1.10; 1.26) | 0.897 | 1.49 (-0.48; 3.46) | 0.133 | 0.43 (-1.13; 1.98) | 0.584 |
| Pictures of facial affect^§^ |  |  |  |  |  |  |  |  |  |  |
| Model 1 | -1.95 (-3.93; 0.04) | 0.054 | 0.19 (-1.27; 1.64) | 0.799 | -0.49 (-1.83; 0.85) | 0.470 | -1.27 (-3.20; 0.65) | 0.188 | -0.86 (-2.45; 0.73) | 0.284 |
| Model 2^†^ | -1.58 (-3.68; 0.52) | 0.135 | 0.12 (-1.33; 1.58) | 0.867 | -0.35 (-1.70; 1.00) | 0.607 | -1.13 (-3.34; 1.08) | 0.307 | -0.56 (-2.14; 1.03) | 0.484 |
| Model 3^‡^ | -1.61 (-3.74; 0.52) | 0.134 | 0.21 (-1.13; 1.55) | 0.756 | -0.32 (-1.65; 1.02) | 0.640 | -1.18 (-3.42; 1.06) | 0.293 | -0.65 (-2.25; 0.95) | 0.418 |

Values are estimates (β) with 95% CI. *Based on multiple linear regression models with the Modified Mediterranean diet scale as continuous variable.

Model 1, unadjusted. ^†^Model 2 adjusted for age, sex and TEI. ^‡^Model 3 adjusted for model 2 plus MWT-B.

Given that few participants failed to finish all cognitive tests, data are only available for ^#^n=74 and n=117 patients with recently diagnosed type 1 and type 2 diabetes, respectively, ^$^n=61 patients with a known type 2 diabetes duration ≥5 years, ^§^n=74 and n=117 patients with recently diagnosed type 1 and type 2 diabetes, respectively and n=60 patients with a known type 2 diabetes duration ≥5 years, ^¥^n=117 patients with recently diagnosed and n=60 patients with a known type 2 diabetes duration ≥5 years and ^§^n=117 patients with recently diagnosed type 2 diabetes.

BACS, Brief Assessment of Cognition in Schizophrenia. MWT-B, multiple choice word test B. TEI, total energy intake. TMT_A/B, trail making test A/B.
